# Associations between maternal social support and stressful life event with ventricular septal defect in offspring: a case-control study

**DOI:** 10.1186/s12884-019-2541-y

**Published:** 2019-11-21

**Authors:** Jiajun Lyu, Kena Zhao, Yuanqing Xia, Anda Zhao, Yong Yin, Haifa Hong, Shenghui Li

**Affiliations:** 1 School of Public Health, Shanghai Jiao Tong University School of Medicine, 227 South Chongqing Road, Huangpu District, Shanghai, 200025 China; 2 Shanghai Children’s Medical Center, Shanghai Jiao Tong University School of Medicine, 1678 Dongfang Road, Pudong District, Shanghai, 200127 China

**Keywords:** Congenital abnormalities, Pregnancy, Social support, Stress, Ventricular septal defect

## Abstract

**Background:**

Previous studies suggested that maternal subjective feeling of stress seemed to be involved in the incidence of congenial heart disease in offspring. To better understand the findings, our study would discuss the relationships of maternal exposure to stressful life event and social support, which are more objective and comprehensive indicators of stress, around periconceptional period with the risk of ventricular septal defect (VSD), the most popular subtype of congenital heart disease.

**Methods:**

A hospital-based case-control study was conducted through June, 2016 to December, 2017. We collected maternal self-reports of 8 social support questions in 3 aspects and 8 stressful life events among mothers of 202 VSD cases and 262 controls. Social support was categorized into low, medium high, and high (higher is better), and stressful life event was indexed into low, medium low, and high (higher is worse). Logistic regression models were applied to estimate adjusted odds ratios and 95% confidence intervals (95% CI).

**Results:**

The adjusted odds ratio of high stressful life event was 2.342 (95% CI: 1.348, 4.819) compared with low stressful life event. After crossover analysis, compared with low event & high support, the adjusted odds ratio of low event & low support, high event & high support, and high event & low support were 2.059 (95% CI: 1.104, 3.841), 2.699 (95% CI: 1.042, 6.988) and 2.781 (95% CI: 1.033, 7.489), respectively.

**Conclusions:**

In summary, we observed an increased risk of VSD when pregnant women exposed to stressful life events, however, social support could, to some extent, reduce the risk of stressful life event.

## **Background**

Within the recent 60 years, the global incidence rate of congenital heart disease (CHD) has increased about 15 times, from 0.6‰ in 1930 to 9.1‰ in 1995 [1]. Similarly, it was reported that the prevalence of CHD has reached up to 11.1‰ in China [2]. Among CHD, ventricular septal defect (VSD) is the most popular subtype, which accounts for more than one fifth of all CHD subtypes [3].

Till now, the etiology of CHD is still unclear. Both genetic factor and environmental exposure are involved in the occurrence of CHD. Previous studies indicated that maternal stress might be an important environmental exposure that could increase the risk of CHD [4, 5]. For example, a hospital-based case-control study in China showed that maternal feeling of stress during pregnancy could bring almost 4 times’ risk (OR = 3.93) of CHD in offspring [4]. Considering maternal feeling stress is a subjective indicator, in order to reduce recall bias, some studies used more objective indicators such as stressful life event (a source of stress) and social support (a potential beneficial buffer against stressor) as alternative evaluation of maternal stress exposure [6–8]. Moreover, compared to taking CHD as a whole, single type of CHD is a better model to explore causes or risk exposure of CHD.

To the best of our knowledge, there were only three studies exploring associations of social support and stressful life event with CHD with single type of CHD [6–8]. The three studies focused on severe CHD, tetralogy of fallot (TOF) and transposition of great arteries (TGA) [6–8], in which the relationship between maternal exposure to stressful life event during pregnancy and the risk of either TOF or TGA was not established, however, social support was found to be associated with a decreased risk of TGA [8]. By contrast, studies among other birth defects, such as neural tube defects **(NTDs), orofacial clefts,** gastroschisis or hypospadias, in general, consistently revealed that social support could decreased the risk, while stressful life event could increase the risk of birth defect [6–11]. Based on the contradictory findings, we speculated the possibility that complex CHD might be more effected genetically. It is imperative to explore the role of maternal stress in single type of simple CHD.

This study was specifically designed to observe the associations of maternal exposure to stressful life event and social support around periconceptional period with the risk of VSD, the most prevalent subtype of simple CHD, in which both individual and combined effect modifications were examined.

## Methods

### Participants

A hospital-based case-control study was conducted in Shanghai Children’s Medical Center through June, 2016 to December, 2017. Sample size was estimated by these formulas [12]:

$$ \mathrm{n}={\left[{Z}_{1-\frac{\alpha }{2}}\sqrt{2\overline{P}\left(1-\overline{P}\right)}+{Z}_{\beta}\sqrt{P_1\left(1-{P}_1\right)+{P}_0\left(1-{P}_0\right)}\right]}^2/{\left({P}_1-{P}_0\right)}^2 $$

$$ \overline{P}=\left({P}_1+{P}_0\right)/2 $$

$$ {P}_1=\left( OR\times {P}_0\right)/\left(1-{P}_0+ OR\times {P}_0\right) $$

As reported in previous studies,12.1–41.1% of pregnant women experienced maternal stress during pregnancy and the odds ratio of maternal stress in CHD was ranged 2.48–3.93 [4, 5, 8]. We used 0.15 and 2.5 as the estimates of *P*_*0*_ and *OR*, respectively. Totally, 152 case. vs 152 controls are minimum sample size to achieve appropriate statistical power (α = 0.05, β = 0.1).

In this study, 202 children with VSD and 262 control children without any birth defects were enrolled. The children in the control group were recruited from the pediatric patients admitted into the same hospital during the same period when the cases were recruited. Among the 262 controls, 132 came from pediatric respiratory medicine, 91 from pediatric general surgery, and 39 from pediatric gastroenterology. To avoid recall bias, all the children were younger than 2 years old.

VSD was defined based on clinical diagnosis and verification by ultrasound. According to the codes of the International Classification of Diseases, Tenth Revision, Clinical Modification, the main VSD types include isolated VSD or VSD with mild complications such as secundum atrial septal defects, coarctation of aorta, patent ductus arteriosus, aortic valve stenosis, and pulmonary stenosis [13]. In the present study, among 202 cases, 85 was isolated VSD, and the other 117 was complicated with secundum atrial septal defects (*n* = 99), coarctation of aorta (*n* = 8), patent ductus arteriosus (*n* = 23), aortic valve stenosis (*n* = 2), or pulmonary stenosis (*n* = 2).

The children with any of the following conditions were excluded from the study: (1) death of mother; (2) mother diagnosed with mental disorder; (3) infant diagnosed with complex CHD (Tetralogy of Fallot, Transposition of great arteries, Hypoplastic left heart syndrome Common truncus, Common ventricle) and (4) inability to locate the mother for interview.

### Procedure

Information on sociodemographic characteristics and parental health-related variables was retrospectively collected through the Parental Behaviors and Environmental Exposure Questionnaire (PBEQ). The women who signed written informed consent to participate and provided consent on behalf of their children were invited to participate in an interview, and to fill in the PBEQ when their children were in hospital. For cases, the interviewed was made after pediatric cardiothoracic surgeons and fetal ultrasonologists had made evaluation and ensured the final diagnosis of CHD. The detailed information of this case-control study has been described elsewhere [14].

The ethical application and consent procedure of this study were approved by the Ethics Committee of Shanghai Jiao Tong University School of Medicine (Approval number: SJUPN-201717).

### Maternal characteristics

Variables regarding maternal characteristic were collected through in-person interview based on PBEQ. Parental ethnic was categorized as Han ethnicity vs. others; maternal age at delivery was grouped into < 35 years vs. ≥ 35 years; maternal educational level was categorized into three groups: middle school and below, high school, and college and above; marital status was grouped as married vs. unmarried/divorced/widowed; maternal residence was categorized as urban vs. suburban/rural; maternal prepregnancy obesity (defined as body mass index > 28.0 kg/m^2^ [15], calculated as weight in kilograms divided by height in meters squared based on prepregnancy weight and height) was categorized as yes vs. no; maternal multiple births was categorized as yes vs. no; infant gender was categorized as male vs. female; family history of CHD was categorized as yes vs. no; maternal prepregnancy diabetes/hypertension was categorized as yes vs. no; maternal smoking/drinking (defined as maternal previous history of smoking and/or drinking) was categorized as yes vs. no; and maternal folic acid use was categorized as yes vs. no.

### Social support and stressful life event evaluation

Social support and stressful life event were assessed by a Social Support and Stressful Life Event Questionnaire (SSSLEQ, as shown Table 4 in Appendix), as a part of PBEQ. The SSSLEQ was developed based on literature review pilot studies [6–9], which includes two sub-scales to collect information regarding social support and stressful life events, respectively. The validity and reliability of SSSLEQ were examined in our sampled participates containing all cases and controls, and the Cronbach’s alpha coefficient was 0.870 for the total questionnaire (0.891 for subscale of social support, and 0.932 for subscale of stressful life event), which indicates that the internal consistency is good and acceptable. The explanatory factor analyses revealed a 5-factor model explaining 58.8% of the total variance.

In subscale of social support, eight questions were used to collect information regarding maternal social support around periconceptional period. The eight questions were conceptually grouped into three aspects: social relationship (three questions), emotional support (two questions), and help with daily tasks (three questions). The response was rated on a 5-scored scale (1 = none, 2 = rarely, 3 = some time, 4 = often, 5 = frequently). The individual social support was defined as “yes” if the response was scored as 4 or 5, and defined as “no” if the response was 0–3. Then the social support index was calculated by summing the count of “yes”, and then was categorized as low, medium high, high when social support index being 0–4, 5–7, and 8 by trisection value in all participants, respectively.

In subscale of stressful life event, eight yes/no questions were applied to collected whether mother experienced the following stressful life events (financial problems, divorce/couple separated, husband violence, lost job, illness/injury of someone close, death of someone close, social relationship difficulty, accident/natural disaster). Each question was scored as 1 if the response was “yes”, or 0 if the response was “no”. The stress index was then calculated by counting the number of “yes”, and was grouped into three levels as low, medium low and high if the index being 0, 1, and ≥ 2 by responded percentile, respectively.

### Statistical analysis

The description of characteristics was made by use of the number and percentage for categorical variables, and Chi-squared test was used to compare differences between groups. Logistic regression analyses were further applied to examine the crude and adjusted associations of social support and stressful life event around periconceptional period with VSD. Adjusted model was controlled for maternal ethnic, maternal age at delivery, maternal education, marital status, residence, maternal prepregnancy obesity, multiple births, infant gender, family history of CHD, maternal smoking/drinking, maternal diabetes/hypertension, and maternal folic acid supplementation. We also examined stressful life event and social support in combination, dichotomizing the social support index score as 0–4 versus 5–8 and the stressful life index score as 0-1versus ≥2 to indicate “low” or “high” level of maternal social support and stressful life events.

In order to achieve consistency in maternal characteristics between cases and controls, the present study adopted a propensity score method matching to balance the characteristic difference between cases and controls [16, 17]. A multivariate logistic regression model was developed to estimate the propensity score, in which all potential confounding variables related to VSD were included in the model. In this procedure, logistic regression was conducted on the group indicator and then uses the resulting propensity variable to select controls for cases. The strength of propensity-score-adjusted analysis lies in taking all covariates along with their interactions as one covariate into account [16, 17]. In propensity-score-matched analysis, controls were matched to cases based on a greedy nearest neighbor matching algorithm on propensity score with a caliper equaling to 0.05.

A statistical significance level was set at *p* value < 0.05. All analyses were performed with the Statistical Package for the Social Sciences (SPSS) (IBM-SPSS Statistics v24.0, Inc. Chicago, IL).

## Results

### Participant characteristics

A total of 464 participants were enrolled in this study (202 VSD cases vs. 206 controls). Characteristics of VSD cases and controls have been shown in Table 1. The differences between VSD cases and controls can be seen in the following three variables: residence, infant gender and folic acid supplementation (all *p* < 0.05). After propensity score matching, all the characteristics was balanced between VSD cases and controls.

**Table 1:** The description of characteristics by VSD vs. Controls (N, %)

	Before Propensity Score Matching			After Propensity Score Matching		
	Controls (*n* = 262)	Cases (*n* = 202)	*p* value	Controls (*n* = 168)	Cases (*n* = 202)	*p* value
Demographic and obstetric characteristics						
Maternal ethnic			0.838			0.759
Han	254, 97.3	194, 97.0		162, 96.4	194, 97.0	
Other	7, 2.7	6, 3.0		6, 3.6	6, 3.0	
Maternal age at delivery			0.915			0.612
< 35 years old	235, 91.1	180, 91.4		155, 92.8	180, 91.4	
≥35 years old	23, 8.9	17, 8.6		12, 7.5	17, 8.6	
Maternal education			0.470			0.833
Middle school and below	57, 22.1	53, 26.8		44, 26.7	53, 26.8	
High school	46, 17.8	36, 18.2		34, 20.6	36, 18.2	
College and above	155, 60.1	109, 55.1		87, 52.7	109, 55.1	
Marital status			0.427			0.296
Married	254, 98.1	196, 99.0		162, 97.6	196, 99.0	
Unmarried/divorced/widowed	5, 1.9	2, 1.0		4, 2.4	2, 1.0	
Residence			0.000			0.537
Urban	163, 62.9	86, 43.2		78, 46.4	86, 43.2	
Suburban /rural	96, 37.1	113, 56.8		90, 53.6	113, 56.8	
Maternal prepregnancy obesity			0.285			0.535
Yes	8, 3.3	3, 1.6		4, 2.6	3, 1.6	
No	237, 96.7	182, 98.4		151, 97.4	182, 98.4	
Multiple births			0.485			0.394
Yes	22, 8.5	13, 6.6		15, 9.0	13, 6.6	
No	238, 91.5	184, 93.4		152, 91.0	184, 93.4	
Infant gender			0.001			0.704
Male	168, 64.1	97, 48.0		84, 50.0	97, 48.0	
Female	94, 35.9	105, 52.0		84, 50.0	105, 52.0	
Family history of CHD			0.074			0.528
Yes	6, 2.3	11, 5.6		5, 3.0	4, 2.0	
No	250, 97.7	187, 94.4		160, 97.0	196, 98.0	
Maternal health indicators and behaviors						
Smoking/drinking			0.823			0.948
Yes	21, 8.9	16, 8.3		13, 7.7	16, 7.9	
No	215, 91.1	177, 91.7		155, 92.3	186, 92.1	
Diabetes/hypertension			0.710			0.438
Yes	33, 12.9	44, 22.7		15, 8.9	23, 11.4	
No	222, 87.1	150, 77.3		153, 91.1	179, 88.6	
Folic acid supplementation			0.044			0.083
Yes	222, 84.7	204, 77.9		142, 84.5	150, 77.3	
No	40, 15.3	58, 22.1		26, 15.5	44, 22.7	

*VSD* Ventricular septal defect

### Social support and stressful life event

The detailed information of social supports and stressful life events by cases vs. controls was shown in Table 2. With respect to social support, control group, compared to cases, more frequently responded “yes” in the following four items, “Did anyone care about you?”, “Did anyone give you emotional support?”, “Did anyone give you a suggestion?”, and “Did anyone help you with your housework?” (all *p* < 0.05). After propensity score matching, except for the response to “Did anyone care about you?”, the responses to other three items were still kept different. While for social support index, difference was existed between cases and controls either before or after propensity score matching (both *p* < 0.05). Regarding stressful life event, compared with controls, cases responded more yes for Financial problem and Husband violence (both *p* < 0.05). After propensity score matching, response to Financial problem was still different. For stressful life event index, difference was shown either before or after propensity score matching (both *p* < 0.05).

**Table 2:** The description of social supports and stressful life events by VSD vs. Controls (N, %)

	Before Propensity Score Matching			After Propensity Score Matching		
	Controls (*n* = 262)	Cases (*n* = 262)	*p* value	Controls (*n* = 168)	Cases (*n* = 202)	*p* value
Social support						
Relationship support						
Chat with someone			0.174			0.222
Yes	173, 66.0	121, 59.9		111, 66.1	121, 59.9	
No	89, 34.0	81, 40.1		57, 33.9	81, 40.1	
Contact with your close friends			0.073			0.051
Yes	178, 67.9	121, 59.9		117, 69.6	121, 59.9	
No	84, 32.1	81, 40.1		51, 30.4	81, 40.1	
Colleague easy to get along with			0.592			0.630
Yes	209, 79.8	157, 77.7		127, 75.6	157, 77.7	
No	53, 20.2	45, 22.3		41, 24.4	45, 22.3	
Emotional support						
Care about you			0.010			0.327
Yes	210, 80.2	141, 69.8		125, 74.4	141, 69.8	
No	52, 19.8	61, 30.2		43, 25.6	61, 30.2	
Give you emotional support			0.008			0.042
Yes	191, 72.9	124, 61.4		120, 71.4	124, 61.4	
No	71, 27.1	78, 38.6		48, 28.6	78, 38.6	
Help with daily tasks						
Give you a suggestion			0.000			0.000
Yes	170, 64.9	90, 44.6		108, 64.3	90, 44.6	
No	92, 35.1	112, 55.4		60, 35.7	112, 55.4	
Colleague help you			0.138			0.101
Yes	199, 76.0	141, 69.8		130, 77.4	141, 69.8	
No	63, 24.0	61, 30.2		38, 22.6	61, 30.2	
Help you with your housework			0.001			0.018
Yes	197, 75.2	123, 60.9		122, 72.6	123, 60.9	
No	65, 24.8	79, 39.1		46, 27.4	79, 39.1	
Social support index			0.020			0.046
0–4 (Low)	68, 26.0	76, 37.6		45, 26.8	76, 37.6	
5–7 (Medium high)	95, 36.3	67, 33.2		64, 38.1	67, 33.2	
8 (High)	99, 37.8	56, 29.2		59, 35.1	56, 29.2	
Stressful life events						
Financial problems			0.001			0.007
Yes	24, 9.5	42, 21.0		17, 10.5	42, 21.0	
No	229, 90.5	158, 79.0		145, 89.5	158, 79.8	
Divorce/couple separated			0.229			0.213
Yes	36, 13.7	36, 17.8		22, 13.1	36, 17.8	
No	226, 86.3	166, 82.2		146, 86.9	166, 82.2	
Husband violence			0.033			0.135
Yes	1, 0.4	7, 3.5		1, 0.6	7, 3.5	
No	253, 99.6	193, 96.5		161, 99.4	193, 96.5	
Lost job			0.307			0.691
Yes	7, 2.8	9, 4.5		6, 3.7	9, 4.5	
No	247, 97.2	189, 95.5		156, 96.3	189, 95.5	
Illness/injury of someone close			0.271			0.189
Yes	18, 7.1	20, 10.0		10, 6.2	20, 10.0	
No	235, 92.9	180, 90.0		152, 93.8	180, 90.0	
Death of someone close			0.391			
Yes	10, 4.0	8, 3.05		5, 3.1	5, 2.5	0.735
No	243, 96.0	195, 97.5		157, 96.9	195, 97.5	
Relationship difficulties			0.120			0.245
Yes	28, 11.0	32, 16.0		19, 11.7	32, 16.0	
No	226, 89.0	168, 84.0		143, 88.3	168, 84.0	
Accident/natural disaster			0.594			0.490
Yes	4, 1.6	2, 1.0		3, 1.9	2, 1.0	
No	250, 98.4	198, 99.0		159, 98.1	198, 99.0	
Stress index			0.013			0.031
0 (Low)	167, 66.0	111, 56.1		106, 65.4	111, 56.1	
1 (Medium low)	60, 23.7	48, 24.2		40, 24.7	48, 24.2	
≥ 2 (High)	26, 10.3	39, 19.70		16, 9.9	39, 19.70	

*VSD* Ventricular septal defect

### The associations between social support/stressful life event and VSD

Table 3 depicts the associations of social support and stressful life event with VSD. It was shown that social support could decrease the risk of VSD, in which the increased level of social support index was related to a declining risk of VSD after controlling for possible confounders (aOR = 0.523 95% CI: 0.283, 0.967 for social support index being medium high; aOR = 0.510 95% CI: 0.321, 0.854 for the index being high). After propensity score matching, the similar tendency was also observed, while the significance occurred merely in the being high.

**Table 3:** Associations of maternal social supports and stressful life events with VSD in offspring

	Before Propensity Score Matching				After Propensity Score Matching		
	Controls *n* = 262	Cases *n* = 202	Crude Model OR (95% CI)	Adjust Model aOR (95% CI)	Controls *n* = 168	Cases *n* = 202	OR (95% CI)
Social support index							
0–4 (Low)	68	76	1	1	45	76	1
5–7 (Medium high)	95	67	0.631 (0.401, 0.992) ^*^	0.523 (0.283, 0.967) ^*^	64	67	0.620 (0.375, 1.025)
8 (High)	99	56	0.533 (0.337, 0.844) ^**^	0.510 (0.321, 0.854) ^*^	59	56	0.592 (0.353, 0.992) ^*^
Stressful life event index							
0 (Low)	167	111	1	1	106	111	1
1 (Medium high)	60	48	1.204 (0.768, 1.886)	1.190 (0.643, 2.201)	40	48	1.146 (0.697, 1.884)
≥ 2 (High)	26	39	2.257 (1.300, 3.916) ^**^	2.342 (1.138, 4.819) ^*^	16	39	2.328 (1.228, 4.414) ^**^
Stressful Life event with social support^a^							
Low stress & High support	178	108	1		113	108	1
Low stress & Low support	49	51	1.715 (1.084, 2.715) ^*^	2.059 (1.104, 3.841) ^*^	33	51	1.617 (0.970, 2.696)
High stress & High support	13	17	2.155 (1.007, 4.612) ^*^	2.699 (1.042, 6.988) ^*^	8	17	2.223 (0.922, 5.364)
High stress & Low support	13	22	2.789 (1.349, 5.765) ^*^	2.781 (1.033, 7.489) ^*^	8	22	2.877 (1.228, 6.739) ^*^

Adjusted Model: adjusted for maternal ethic, maternal age at delivery, maternal education, marital status, residence, maternal prepregnancy obesity, multiple births, infant gender, and family history of CHD, maternal smoking/drinking, maternal diabetes/hypertension, maternal folic acid supplementation

*aOR* adjusted odds ratio *CI* confidence interval *OR* odds ratio *VSD* Ventricular septal defect

^a^ Women were designated as having low event if the life events index score was 0–1 and as having high event if it was ≥2. Women were designated as having low social support if the social support index was 0–4 and as having high support if it was ≥5

* *p* value < 0.05; ** *p* value < 0.01

By contrary, stressful life event could increase the risk of VSD. Higher level of maternal stressful life event might increase the risk of VSD in offspring. The risk gradually increased when the maternal exposure to stressful life event was rising from medium low (OR = 1.204 95% CI: 0.768, 1.886) to high (OR = 2.257 95% CI: 1.300, 3.916). In the adjusted model, the trend was similarly found (aOR = 1.190 95% CI: 0.643, 2.201 for stressful life event index being medium low; and aOR = 2.342 95% CI: 1.138, 4.819 for index being high). When the analyses were repeated after propensity score matching, very similar results were obtained.

**Fig. 1:**
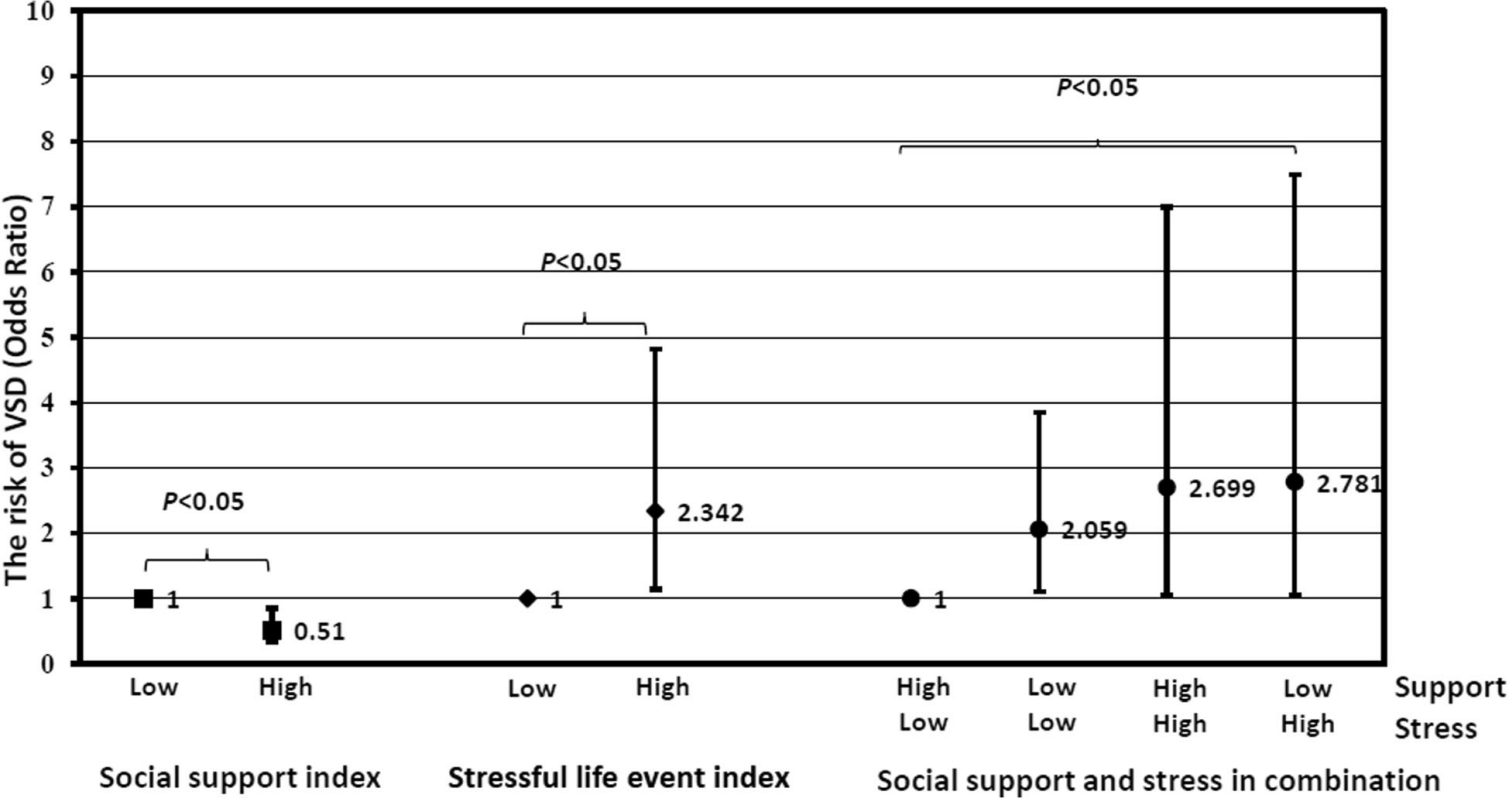
Social support & stressful life event with VSD. Associations of maternal social support and stressful life events around periconceptional period with the risk of VSD in offspring

According to the crossover analysis of social support and stressful life event (as shown in Fig. 1), it seemed that social support could reduce the risk of stressful life event. Compared to maternal exposure to low event & high support, exposure to low event & low support and high event & high support could increase the risk of VSD by 71.5% (OR = 1.715 95% CI: 1.084, 2.715) and 115.5% (OR = 2.155 95% CI: 1.007, 4.612), respectively. Maternal exposure to high event & low support has the greatest risk (OR = 2.789 95% CI: 1.349, 5.765). Further adjusting for maternal characteristics, the protective effect modification of high social support still lied there (low event & low support: aOR = 2.059 95%CI: 1.104, 3.841; high event & high support: aOR = 2.699 95% CI: 1.042, 6.988; high event & low support: aOR = 2.781 95% CI: 1.033, 7.489). After propensity score matching, the protective tendency also can be seen, although the significance appeared just in the group of high event & low support.

## Discussion

Previous study indicated that maternal stress around periconceptional period could be involved in the incidence of CHD in offspring [4, 5]. However, the findings were not always consistant and, in some cases, cannot be repeated in single type of CHD [6–8]. Considering most previous studies mainly focused on severe CHD, such as TOF and TGA, which might be caused by more complicated factors. This study is the first to analyze the role of maternal exposure to social support, stressful life events and their interactions in the risk of VSD, the most prevalent subtype of simple CHD. We found that maternal social support around periconceptional period was associated with a decreased risk of VSD, in which some dose-effect relation was observed that the higher maternal social support the pregnant women got the lower risk of VSD their children would have. It was also demonstrated that maternal exposure to more stressful life events was associated with an increased risk of VSD. The crossover analysis further revealed that social support could reduce the risk of stressful life event.

As our knowledge, up to date only three previous studies explored the risk of maternal exposure to stressful life event in the incidence of TOF and TGA, however, none of them established the relationship [6–8]. By contrast, we found that maternal exposure to two or more stressful life events could increase the risk of VSD in offspring. TOF and TGA, compare to VSD, have more complicated cardiovascular malformations, we speculate that more grave exposure, gene defects/abnormalities, and their interaction would be implicated in the triggering of defects [18]. The other potential explanation could be that, compared to the three studies, this study collected different and more stressful life events. We did observe a tendency toward higher risk with more exposure to stressful life events. The three studies mentioned above provided some support for our speculation. For example, one of which using data from National Birth Defects Prevention Study, the USA, demonstrated a high risk of TOF (OR = 3.1) among those women who experienced 6–7 stressful life events, although 95% confidence interval included one (0.8–12.2) [8]. However, maternal exposure to stressful life events less than 6 didn’t show the risk of TOF (OR ranged from 0.7 to 1.1) [8]. Considering only 3 women exposed to 6–7 stressful life events during periconceptional period among 311 TOF cases, we assume that the effect modification size would be statistically significant when enlarging the sample size. Some other studies suggested that subjective mental stress around periconceptional period was associated with an increased risk of CHD, in which the maternal stress was dichotomized into yes or no [4, 5]. We found that the impact of stressful life event was smaller than that of subjective mental stress, however, when it came to particular stressful life event such as bereavement, the effect modification size appeared to be similar to our study. A registry-based study in Denmark reported that prenatal exposure to bereavement increased the risk of CHD by 1.4 times (OR = 2.4 95% CI: 1.4, 4.2) in offspring [19].

The protective effect modification of social support on VSD in our study was quite similar to one previous study in TGA [8]. Both of them revealed that good social support and assistance around periconceptional period would be helpful to reduce the risk of abnormal cardiac morphogenesis by approximately 50%. A number of studies conducted in other birth defect also provided evidence that more social support was associated with reduced risk of birth defects, including NTDs, gastroschisis and hypospadias [8, 9, 11]. When examing the combinated influence of stressful life events and social support, our findings suggested that women who exposed to highest stressful life events and lowest social support during periconceptional period had the largest risk estimation for VSD; and more social support could mildly modify the association.

Taken our findings together, those pregnant women who experienced stressful events during periconceptional period should be taken high priority in promoting antenatal care since undesirable and uncontrollable negative experience could increase the risk of VSD in offspring. In daily life, the most important effort is to improve family and social support, help them get more love, assistance and encouragement in emotion and financial condition. To give a good birth and good care of the new baby it is important for obstetricians to evaluate maternal social support and stressful life events during prenatal examinations. They might alert the pregnant women of high mental stress and low social support to their children’s cardiovascular malformations.

The exact mechanisms underlying the role of social support or stressful life event in CHD or VSD are still unknown. Several mechanisms were proposed, including thrombotic, inflammatory, or endocrine pathways. For example, it was reported that exogenous corticosteroid use during pregnancy could pose a small increased risk of birth defects [20]. There might be the possibility that increased production of corticosteroids in response to maternal stress exposure may play a role in VSD of offspring [21]. Moreover, there is an increasing evidence for the transgenerational impact of early-life experiences and the involvement of epigenetic pathways in these effect modifications [22].

The strengths of our study included its specific cases choosing the most frequent single type of CHD, more detailed information regarding social support and stressful life events, dose-effect assessment of social support and stressful life event, stratify analysis of stress and social support in combination, adjustment for several potential confounders, and propensity score matching to balance differences between cases and controls. Our examinations of social support and stressful life event were much more comprehensive (a total of 16 questions) than any of previous studies exploring associations of stress with birth defects (up to 10 questions) [8, 9]. The biggest strength of our study is that social support may provide a beneficial buffer against the negative impact of stress, but few other studies took it into consideration, and the results were not as impressive as ours [7–9]. Considering the possible selection bias and recall bias, we limited the children to 2 years old, however, most other studies did not do better, for example, a previous case-control study limited the children to 7 years old [4]. Meanwhile, we particularly chose to focus on questions related to concrete major life events rather than subjective feeling of maternal stress.

However, our study still has some limitations. We did not particularly focus on specific stressful event, and there is the possibility that some types may be more stress-inducing. Recall bias and selection bias are inevitable in a case-control study. A hospital-based study has inherent weaknesses, since hospital-based cases could not represent the total distribution of CHD occurring in the local population. Although after propensity score matching, we balanced the residence of the enrolled family. However, as a result of sample restrict, we merely matched 168 controls. Due to these limitations, the findings require further confirmation by more studies with larger sample size or prospective longitudinal design on particular stress event.

## Conclusions

This study, for the first time, observed an increased risk of VSD among mothers who reported more stressful life events and a decreased risk among mothers who got more social supports around periconceptional period. Moreover, social support could reduce the risk of stressful life event. The impact of maternal social support and stressful life events on risks of CHD has been studied much less than the impact on risks of other birth defect, such as NTDs [8]. Against the background that CHD has been the most prevalent birth defect, and in which VSD places the first, the findings of our study have important clinical and public health implications for the control of birth defects. Due to the retrospective design of our study, prospective longitudinal studies are needed to provided further and enriched evidence.

## Data Availability

The datasets analyzed during the current study are not publicly available due to the protection of patients’ information but are available from the corresponding author on reasonable request.

## Appendix

**Table 4:** Social Support and Stressful Life Event Questionnaire (SSSLEQ)

Social support	None	Rarely	Some time	Often	Frequently
Relationship support					
Did you often chat with someone?					
Did you often Contact with your close friends?					
Did your colleague easy to get along with?					
Emotional support					
Did anyone care about you?					
Did anyone give you emotional support?					
Help with daily tasks					
Did anyone give you a suggestion?					
Did your colleague help you?					
Did anyone help you with your housework?					
Stressful life events	No		Yes		
Financial problems					
Divorce/couple separated					
Husband violence					
Lost job					
Illness/injury of someone close					
Death of someone close					
Relationship difficulties					
Accident/natural disaster					

Note: A series of questions about social support and stressful life events were asked in separate sections of the questionnaire. From these questions, we determined whether mothers had received social support and experienced stressful life events during the periconceptional period

## Abbreviations

aOR: Adjusted odds ratio
CHD: Congenital heart disease
CI: Confidence interval
NTDs: Neural tube defects
OR: Odds ratio
PBEQ: Parental behaviors and environmental exposure questionnaire
SSSLEQ: Social support and stressful life event questionnaire
TGA: Transposition of great arteries
TOF: Tetralogy of fallot
VSD: Ventricular septal defect
